# Effect of the screw-retained abutment material on the fatigue behavior of lithium disilicate restorations

**DOI:** 10.1590/0103-644020256936

**Published:** 2026-02-02

**Authors:** Eduarda de Miranda Bohrer, Pablo Machado Soares, Maria Gabriela Packaeser, Lucas Saldanha da Rosa, João Paulo Mendes Tribst, Cornelis Johannes Kleverlaan, Luiz Felipe Valandro, Gabriel Kalil Rocha Pereira

**Affiliations:** 1 Faculty of Dentistry, Federal University of Santa Maria (UFSM), Santa Maria, Rio Grande do Sul State, Brazil; 2 Post-Graduate Program of Oral Sciences, Federal University of Santa Maria (UFSM), Santa Maria, Rio Grande do Sul State, Brazil; 3 Department of Reconstructive Oral Care, Academic Centre for Dentistry Amsterdam (ACTA), Universiteit van Amsterdam and Vrije Universiteit, Amsterdam, North Holland, The Netherlands; 4 Department of Dental Materials Science, Academic Centre for Dentistry Amsterdam(ACTA), Universiteit van Amsterdam and Vrije Universiteit, Amsterdam, North Holland, The Netherlands

**Keywords:** ceramic materials, implant-supported restorations, abutment materials, mechanical behavior, prosthodontics

## Abstract

The aim of the study was to evaluate the effect of the screw-retained abutment material (zirconia (Yz), lithium disilicate (Ld), Polymer-infiltrated ceramic network (Picn), and Polietheretherketone (Peek)) on the fatigue behavior and stress distribution of lithium disilicate restorative material. Ld discs (Ø= 10 mm; thickness = 1 mm) were obtained and allocated into four groups according to the abutment material factor: Yz, Ld, Picn, and Peek (Ø= 10mm; thickness 3 mm). A screw access hole was made at the center of each abutment and filled with resin composite (thickness = 2 mm). The materials were adhesively bonded and tested under monotonic mechanical load (n= 3) and cyclic fatigue (n= 15; initial load: 200 N for 10,000 cycles; step size: 100 N/10,000 cycles; frequency: 20 Hz) until failure. Finite element and Scanning Electron Microscopy (SEM) analyses were also performed. The obtained data were analyzed by 1-Way ANOVA for monotonic (α=0.05) and Kaplan Meier and log-rank post-hoc for fatigue data tests. All groups presented a decrease in the fatigue failure load (FFL) when compared to the monotonic test. The abutment material affected the lithium disilicate restoration performance (*p*=.048; F= 4.134), since zirconia showed the highest FFL (*p*<.05), followed by Picn and Ld, which were similar to each other (*p*>.05). The lowest values of FFL were found for Peek (*p*<.05). The screw-retained abutment material influences the fatigue behavior of lithium disilicate restorations, being zirconia the most indicated material to enhance mechanical performance.

## Resumo

O objetivo deste estudo foi avaliar o efeito do material do pilar parafusado (zircônia (Yz), dissilicato de lítio (Ld), rede cerâmica infiltrada por polímero (Picn) e polieteretercetona (Peek)) no comportamento à fadiga e na distribuição de tensões do material restaurador de dissilicato de lítio. Discos de Ld (Ø = 10 mm; espessura = 1 mm) foram obtidos e alocados em quatro grupos de acordo com o material do pilar: Yz, Ld, Picn e Peek (Ø = 10 mm; espessura = 3 mm). Um orifício de acesso para o parafuso foi feito no centro de cada pilar e preenchido com resina composta (espessura = 2 mm). Os materiais foram adesivamente cimentados e testados sob carga mecânica monotônica (n = 3) e fadiga cíclica (n = 15; carga inicial: 200 N por 10.000 ciclos; incremento: 100 N/10.000 ciclos; frequência: 20 Hz) até a falha. Análises de elementos finitos e microscopia eletrônica de varredura (MEV) também foram realizadas. Os dados obtidos foram analisados ​​por ANOVA-1 fator para o teste monotônico (α = 0,05) e por Kaplan-Meier e teste post-hoc de log-rank para os dados de fadiga. Todos os grupos apresentaram uma diminuição na carga de falha por fadiga (CFF) quando comparados ao teste monotônico. O material do pilar influenciou o desempenho da restauração de dissilicato de lítio (p = 0,048; F = 4,134), sendo que a zircônia apresentou a maior FFL (p < 0,05), seguida por Picn e Ld, que foram semelhantes entre si (p > 0,05). Os valores mais baixos de FFL foram encontrados para o Peek (p < 0,05). O material do pilar parafusado influencia o comportamento de fadiga das restaurações de dissilicato de lítio, sendo a zircônia o material mais indicado para melhorar o desempenho mecânico.

**Figure:**
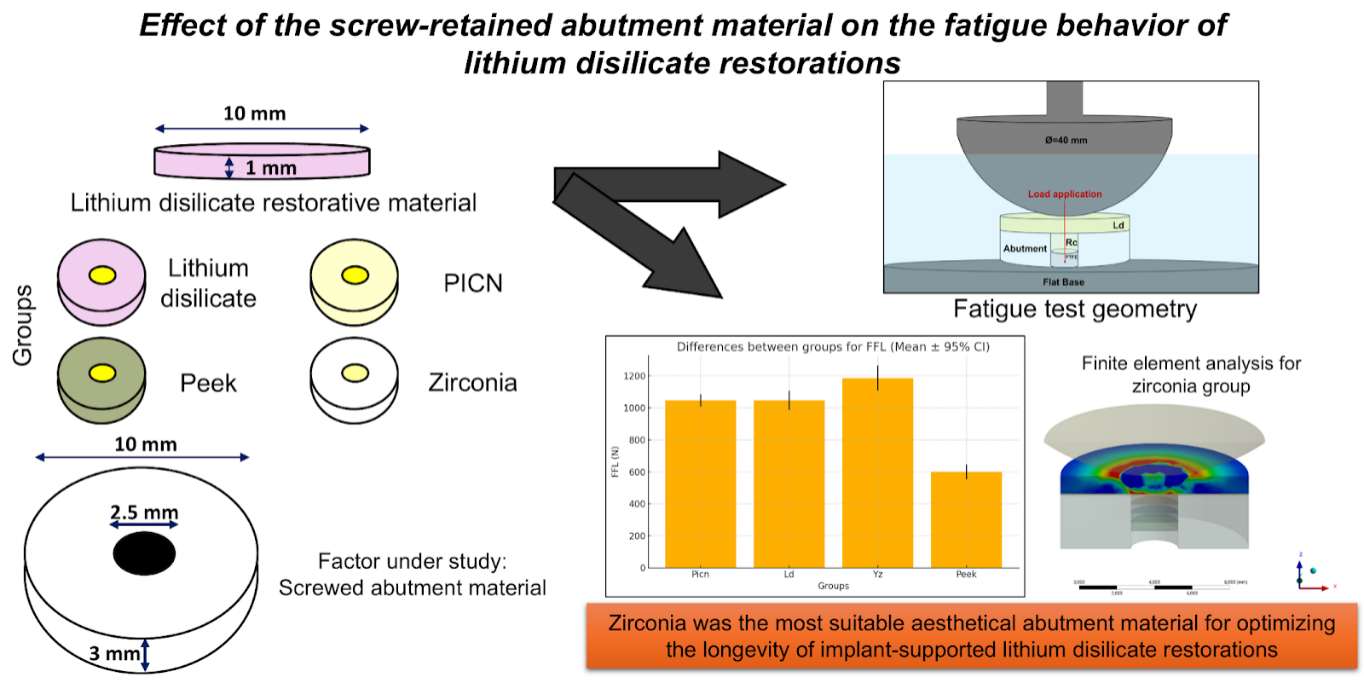

## Introduction

Ceramic materials are widely used in oral rehabilitation with implant-supported crowns, considering their excellent aesthetic results, mechanical performance, bonding potential, and biocompatibility ^1^ ^,^ ^2^. In this sense, lithium disilicate-reinforced glass ceramic stands out due to its versatility, combining excellent mechanical properties, color stability, and translucency ^3^ ^,^ ^4^. These characteristics are given by the microstructure of lithium disilicate, which is composed of crystalline content, surrounded by a 70% volume fraction of silica matrix, thus making this material an excellent option for several applications, such as inlays, onlays, full-crowns, and even hybrid implant abutments ^3^ ^,^ ^5^ ^,^ ^6^.

Considering implant-supported restorations, there are several available systems for clinical practice. Regarding the implant abutment connections, these may be luted on the implant, screw-retained onto it, or even be a one-piece system, where the abutment and implant are one single component ^7^ ^,^ ^8^. When screw-retained abutments are adopted, a screw access hole is present, which is usually filled with polytetrafluoroethylene (PTFE) tape ^9^ ^,^ ^10^, or even the combination of PTFE tape and resin composite to cover and protect the screw top surface ^10^ ^,^ ^11^. One important advantage of using screw-retained abutments is the reversibility of the system by removing the screw, which allows modifications and replacements when necessary. Even though both systems are widely used in clinical practice, previous studies showed that the presence of screw access hole and the filling protocol affect the mechanical behavior in fatigue and stress distribution along the restorative material when considering titanium and zirconia abutment materials ^11^ ^,^ ^12^. Hence, not only the crown properties, but also the abutment material condition dictate the mechanical performance and longevity of the entire restorative set.

When more aesthetic abutments are required, some options are indicated as alternatives to conventional titanium components, as zirconia and lithium disilicate ceramic, which are considered two of the most resistant options among dental ceramics ^13^ ^,^ ^14^. Polymeric materials, such as polyetheretherketone (Peek), offer promising alternatives for abutments due to their high biocompatibility, satisfactory mechanical strength, cost-effectiveness, and lower elastic modulus compared to ceramic materials ^15^ ^,^ ^16^. Additionally, polymer-infiltrated ceramic network (PICN) materials offer high fracture toughness, which may reduce crack propagation in restorations ^17^ ^,^ ^18^. Previous studies showed that more rigid abutment materials reduce the bending effect of the luted crown material over it, thus concentrating more stress and increasing the load to failure of the restorative set ^19^. When the screw access hole is present at the center of the abutment material, it can be assumed that the presence of the hole and a hole-filling material may affect the abutment support and thus reflect on the mechanical behavior of the restorative material ^12^. However, there is still no previous study evaluating such a condition for different abutment materials. Thus, it is essential to verify the relevance of such a factor when considering screw-retained abutment scenarios, in order to determine the best restorative approach when aesthetic screw-retained abutments are required.

Therefore, the aim of the present study was to evaluate the effect of the abutment material (zirconia, lithium disilicate, Picn, and Peek) on the mechanical behavior in fatigue and stress distribution of lithium disilicate restorative material, in the presence of a filled screw access hole in the abutment. The null hypothesis was that the abutment material would not impact the load for failure and stress distribution of the restorative lithium disilicate.

## Materials and methods

### Study design

The adopted materials, as well as their batch number and composition, are described in Table 1. Besides, the experimental design is depicted in Table 2. The factor under study consists of the abutment material (lithium disilicate, Peek, polymer-infiltrated ceramic network (PICN), and zirconia) for implant-supported restorations (lithium disilicate), which were luted on the abutment material and tested under monotonic test (n=3) and cyclic fatigue test (n=15). The sample size determination was based on several previous studies that adopted such mechanical tests and evaluated materials with similar microstructures and geometry ^12^ ^,^ ^14^ ^,^ ^20^ ^,^ ^21^ ^,^ ^22^. For all groups, a screw-access hole was present at the center of each abutment material, filled with bulk-fill resin composite. A simplified specimen geometry was adopted (three-layer assembly: 1 mm restorative disc; resin cement; and three mm-thick abutment disc; Ø = 10 mm), which was widely used and described by previous studies ^12^ ^,^ ^20^ ^,^ ^21^ ^,^ ^22^.

**Table 1:** Materials used in this study, their commercial name, manufacturers, batch number and main composition.

Material	Commercial Name	Manufacturer (Batch number)	Composition
Yttria-stabilized tetragonal zirconia polycrystal (Yz)	IPS e. max Zircad MO	Ivoclar AG (Lot. T18593)	88.0 - 95.5 wt% ZrO_2_, > 4.5 - ≤ 6.0 wt% Y_2_O_3_, ≤ 5.0 wt% HfO_2_, ≤ 1.0 wt% Al_2_O_3_, max 0.02% SiO_2_, 0-0.3% Fe_2_O_3_
Polyetheretherketone (Peek)	Ceramill PEEK	Juvora (Lot. J000142)	90 wt% Colourless organic thermoplastic polymer in the polyaryletherketone family, used in engineering applications, 10% TiO_2_
Polymer infiltrated ceramic network (Picn)	VITA Enamic; 2 M2-HT EM-14	VITA Zahnfabrik, (Lot. 52060)	Al_2_O_3_, 9-11% Na_2_O, 4-6% K_2_O, 0.5-2% B_2_O_3_, <1% ZrO_2_, <1% CaO); 14% organic polymer (UDMA-urethane dimethacrylate, TEGDMAtriethylene glycol dimethacrylate).
Lithium disilicate glass-ceramic (Ld)	IPS e.max CAD; LT A2/C 16	Ivoclar AG (Lot. Z039GR)	58-80% SiO_2_, 11-19% Li_2_O, 0-13% K_2_O, 0-8% ZrO_2_, 0-5% Al_2_O_3_.
Cement	Multilink N Transparent	Ivoclar AG (Lot. Z00JPZ)	Dimethacrylates, HEMA, barium glass filler, Ba-Al-Fluoro-Silicate glass, ytterbium trifluoride, highly dispersed silica, catalysts and stabilizer, pigments
Primer	Monobond N	Ivoclar AG (Lot. Z040SJ)	Alcohol solution of silane methacrylate, phosphoric acid methacrylate, and sulphide methacrylate

**Table 2:** Study design describing the experimental groups (based on the factor abutment material), their screw-access filling protocol, luted restorative material and performed tests.

Groups codes	Abutment material	Screw-access hole filling	Resin cement	Restorative material	Tests
Picn	Polymer-Infiltrated ceramic network (VITA Enamic, VITA Zahnfabrik)	2 mm filling with a Bulk fill resin composite (Tetric N-ceram bulk fill, Ivoclar AG) + 1 mm filling with a tape simulating the protection of the screw head	Multilink N, Ivoclar AG	Lithium disilicate (IPS e.max CAD, Ivoclar AG)	Monotonic test (n=3) Cyclic fatigue (n=15) Finite element analysis Fractography
Ld	Lithium disilicate (IPS e.max CAD, Ivoclar AG)				
Yz	Yttrium stabilized zirconia (IPS e.max ZirCAD MO, Ivoclar AG)				
Peek	Ceramill PEEK, Juvora				

### Abutment Material Preparation

To obtain the abutment material specimens, discs of Yz (IPS e.max ZirCAD MO, Ivoclar AG, Schaan, Liechtenstein), Peek (Ceramill PEEK, Juvora, Lancashire, United Kingdom), Picn (VITA Enamic, VITA Zahnfabrik, Bad Sackingen Germany) and lithium disilicate (Ld - IPS e.max CAD, Ivoclar AG, Schaan, Liechtenstein) were prepared by grinding CAD/CAM (Computer-Aided Design/Computer-Aided Manufacturing) blocks in a grinding machine (EcoMet/AutoMet 250, Buehler, Lake Bluff, USA) until a cylindric shape was obtained (Ø = 10 mm for all materials but zirconia, which was ground in 12 mm, considering its sintering shrinkage of 20% according to the manufacturer). Then, the cylinders were cut into slices (3.85 mm of thickness for zirconia, 3.10 mm for other materials) on a precision cutting machine (IsoMet 1000, Buehler, Lake Bluff, USA) under constant water irrigation and polished with silicon carbide (SiC) sandpaper in grits #400, #600 and #1200 (3M, Sumaré, Brazil). In order to prepare the screw access hole, the discs were cleaned and attached to a preparing device ^23^, then the preparation was carried out by using a cylindrical diamond tip (4102MF, KG SORENSEN, Cotia, Brazil) attached to a multiplier piece of high-speed (T3 Line E 200 handpiece up to 170,000 rpm, Sirona, Bensheim, Germany), introduced perpendicularly into the discs to create a first access channel. Then, a second diamond tip (3101FF, KG SORENSEN) was used to give the hole the appropriate contour and diameter (Ø = 3.12 mm for pre-sintered zirconia; 2.5 mm for all other materials). After that, the discs were sintered/crystallized in a sintering furnace according to the demand of each material (except for Peek and Picn) and the manufacturer's recommendations.

### Restorative material preparation

The restorative material was prepared from CAD/CAM blocks of lithium disilicate by using similar methodological steps described for the abutment material preparation. Thus, after the preparation, pre-sintered glass-ceramic discs were obtained with a thickness of 1 mm. Considering the adopted restorative assembly, these specimens were prepared without the presence of a screw access hole, thus simulating luted restorations over screw-retained abutments. After the preparation of the restorative discs, all samples were randomized into four experimental groups, considering the different abutment materials (Table 2).

Considering that the CAD/CAM milling process modifies the surface topography and increases the roughness of dental materials ^24^, an in-Lab simulation of the CAD/CAM roughness was performed on the bonding surface of lithium disilicate restorative material. The simulation was performed by a trained researcher, who marked the restorative ceramic in 2 axes (x and y) with a marking pen, followed by manual grinding with 100 × 50 mm #60 SiC paper under constant water irrigation (one SiC paper for each specimen). Light digital pressure was applied to grind the specimen for 15 s on each axis ^25^. After that, six measurements of Ra and Rz were carried out for each specimen (3 for each axis) using a profilometer (Mitutoyo SJ-410, Mitutoyo Corporation, Kawasaki, Japan). After the measurements, a roughness pattern similar to those mentioned by a previous study, which used CAD/CAM milling to manufacture samples, was found, with a Ra of approximately 1.8 for the glass-ceramic tested in our study ^24^.

Finally, the lithium disilicate specimen was crystallized according to the manufacturer’s recommendation (840 °C, 7 min vacuum, Vacumat 6000 MP, VITA Zahnfabrik, Bad Sackingen, Germany).

### Screw access filling

After the abutment specimen’s preparation, the filling of the access hole for the simulated screw was carried out. The abutment discs were positioned on a glass plate, with the bonding surface facing down, and then a bulk fill resin composite (Tetric N-ceram bulk fill, Ivoclar AG, Schaan, Liechtenstein) was applied in a single increment and condensed inside the hole of each abutment material, in order to control the thickness with a millimeter probe (2 mm). After the application of the increment, the resin composite was photoactivated at 1200 mW/cm2 (Radii-cal LED curing light, SDI, Bayswater, Australia). After that, PTFE tape was added to fill the remaining abutment hole, simulating the clinical scenario.

### Bonding procedures and cementation

The prepared materials were cleaned in an ultrasonic bath (1440 D - Odontobras, Ribeirão Preto, Brazil) with 78% isopropyl alcohol for 5 min. Then, surface treatments were performed according to the microstructure and manufacturer's recommendation for each adopted material. For Peek and Yz abutment materials, the bonding surface was air-abraded with aluminum oxide particles (45 μm particle size, Polidental) for 10 s at a distance of 1 cm with 2.8 bar pressure and oscillatory movements ^14^. Then, a primer containing MDP (10-Methacryloyloxydecyl dihydrogen phosphate) and silane (Monobond N, Ivoclar AG, Schaan, Liechtenstein) was applied for 60 seconds on both materials (Peek and Yz), followed by gently air-drying ^26^.

The bonding surfaces of PICN and lithium disilicate (abutment and restorative material) specimens were acid-etched with 5% hydrofluoric acid (Condac Porcelana 5%, FGM, Joinville, Brazil), for 20 s for lithium disilicate and 60 s for PICN. Then, all specimens were washed by air-water spray and cleaned with isopropyl alcohol in an ultrasonic bath, followed by the primer application (Monobond N, Ivoclar AG) for 60 seconds.

After the surface treatments, the resin cement pastes (Multilink N, Ivoclar AG, Schaan, Liechtenstein) were mixed and applied to the restorative material, which was then placed over its respective abutment material. A load of 2.5 N was applied to the middle of the top surface of the specimens, and the excess cement was removed with a microbrush ^14^. The resin cement was light-activated at 1200 mW/cm2 (Radii-cal LED curing light, SDI) around all sides of the specimen (0°, 90°, 180°, 270°) and on the top surface for 20 s for each exposure. After the luting procedures, the specimens were stored in distilled water (37 °C) for at least 24 hours before the fatigue tests.

### Monotonic and cyclic fatigue mechanical tests

First, a monotonic mechanical test (n=3) was performed in order to determine the nominal load for the failure of the bonded set (restorative ceramic/resin cement/abutment material) according to each group (Table 2). The test was performed in a universal machine (DL 2000; EMIC, São Paulo, Brazil) using a loading cell of 1000 Kgf, with a crosshead speed of 0.5 mm/min, until the first crack was noted. The obtained data were used to determine the fatigue parameters and for posterior registration of the decrease of load for failure from monotonic to cyclic fatigue behavior.

After the monotonic test was finished, a cyclic fatigue test (n=15) was performed (ElectroPuls E3000, Instron Corporation, Norwood, USA) through the use of a stainless-steel piston (Ø=40 mm) and a flat metal base where the specimen was stabilized. The test was performed under distilled water, and with an adhesive tape (110 μm) placed on the top surface of the specimen to improve its contact with the piston ^27^. The following parameters were adopted (initial load: 200N for 10,000 cycles; step size: 100N/10,000 cycles; frequency: 20Hz), and the test was carried out until the failure of the restorative material (first crack), which was checked at each step by transillumination. The fatigue failure load (FFL) and cycles for failure (CFF) were then collected for statistical purposes.

### Scanning Electron Microscopy (SEM) analysis

After the cyclic fatigue test, the failed restorative material was carefully detached from its respective abutment to be inspected in a stereomicroscope (Discovery V20, Carl Zeiss, Gottingen, Germany) with a specific lens (Achromat S 0.5 × FWD 151 mm, Carl Zeiss, Gottingen, Germany) at 30×, 200× and 500× magnification to assess the failure pattern according to each group. Representative samples of each group were selected (n=1), gold-sputtered, and analyzed by scanning electron microscopy (VEGA3, Tescan) to determine crack origin ^28^.

### Finite element analysis

**Figure 1:**
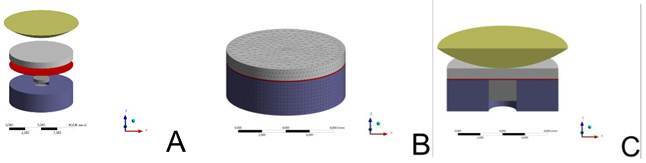
Description of test geometry for finite element analysis. A: Specimen geometry for each layer (abutment, resin composite, resin cement, lithium disilicate restorative material, and load applicator); B: Mesch arrangement; C: Test geometry for in-silico analysis, mimicking in vitro fatigue test.

In order to determine the maximal principal stress (MPa) within the lithium disilicate restorative material according to the abutment material factor, a three-dimensional (3D) finite element analysis was performed. For that, 3D geometric models consisting of the lithium disilicate restorative material, the steel piston, and the four abutment material discs (Peek, Picn, Ld, and Yz) were obtained according to the in vitro mechanical test (Figure 1). The Rhinoceros software program (CAD Rhinoceros 6.0 software, McNeel North America, Seattle, USA) was used to carry out the finite-element modeling. Young’s moduli (GPa) and Poisson’s ratios of the tested materials were obtained from the materials brochures and previous studies Yz (E = 210 GPa; v = 0.31), Peek (E = 4.1 GPa; v = 0.4), Picn (E = 30 GPa, v = 0.28), Ld (E = 95 GPa; v = 0.25), bulk-fill resin composite (E = 10 GPa; v = 0.3), resin cement (E = 7.5 GPa, v = 0.3) and stainless steel (E = 190 GPa; v = 0.27) (14,29-31). A digital load (300 N) was applied to the top surface of the cut steel ball. The connections within the bonded sets were considered perfectly bonded for all materials, except for the steel piston/restoration relation, which was considered frictional. The nodes at the top surface of the cut steel ball were fixed in the horizontal plane, allowing movement in the vertical dimension only. A linear static structural analysis was applied, where all materials were considered isotropic, linear, and homogeneous. The stress distribution analysis was performed using ANSYS software CAE (ANSYS 19, ANSYS Inc., Houston, USA), and the Maximum Principal Stress was used as the analysis criterion.

### Statistical analysis

In order to evaluate the obtained data, first normality and homoscedasticity analysis were performed through the Shapiro-Wilk and Levene tests, respectively. Considering the usual assumption of the data, One-Way analysis of variance and Tukey (post-hoc) tests were performed to determine the effect of the factor under study (abutment material) during the fatigue tests. Besides, a survival analysis was performed using the Kaplan-Meier test and Mantel-Cox post-hoc (log-rank) tests (α=.05) with the SPSS version 21 statistical program (IBM, Chicago, IL, USA).

## Results

The results of the mechanical tests (monotonic and cyclic fatigue) are depicted in Table 3. One-way ANOVA showed that the filled abutment material presented a significant effect on the mechanical behavior of lithium disilicate restorative material (*p*= 0.048; F= 4.134). The Kaplan Meier analysis for fatigue test corroborated such results, since Yz abutment showed the highest failure load and cycles for failure means, followed by Picn and Ld abutment materials (*p*<0.05). The lowest values of FFL and CFF were found for Peek (*p*<0.05).

The survival rates align with these findings, which can be noticed when considering the step of 1100N, where Yz presented 53% survival probability, while Picn and Ld presented 7% and 20%, respectively (Table 4). For the Peek abutment, all specimens failed after the 800N step.

**Figure 2:**
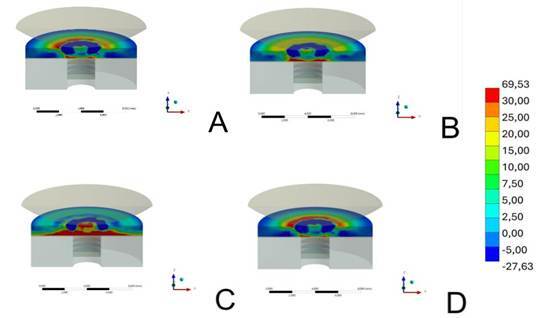
Finite element analysis according to abutment material factor (A-Ld; B-Picn; C-Peek; D-Yz) bonded to lithium disilicate restorative material. Higher tensile stress (red-orange zone) was present at the bonding surface of the restorative material when bonded to the Peek abutment. On the other hand, tensile stress was lower when bonded to the YZ foundation substrate, while more stress was concentrated at the top surface.

The maximal principal stress of each group is depicted in Table 3 and Figure 2. The finite element analysis showed that higher stress concentration was noted at the bottom surface (tensile side) of the lithium disilicate restorative material when bonded to Peek abutment material, followed by Picn and Ld groups, respectively. The Yz presented the lower value of the Maximal principal stress peak, showing more stress concentration at the top surface of the restorative material, mainly when compared to the Peek and Picn groups.

**Table 3:** Results of mechanical tests presenting mean, standard deviation (SD), 95% confidence intervals (95% CI), of fatigue failure load (FFL), number of cycles for failure (CFF), monotonic load to failure outcomes, percentage of decrease from monotonic to fatigue tests and Maximum Principal Stress peak per restorative material.

Groups	FFL (in N)*	CFF (Counts)*	Monotonic Load to Failure (in N)	% of load decrease from monotonic to fatigue tests	Maximum Principal Stress peak (MPa)
	Mean (95% CI)	Mean (95% CI)	Mean (SD)		
Picn	1047 (1009 - 1084)^B^	94,159 (90,105 - 98,213)^B^	1526 (1104)	31	29.54
Ld	1047 (986 - 1106)^B^	89,866 (77,089 - 102,643)^B^	1715 (516)	39	23.72
Yz	1186 (1108 - 1265)^A^	108,666 (100,811 - 116,522)^A^	2580 (113)	54	22.16
Peek	600 (553 - 646)^C^	48,231 (43,171 - 53,290)^C^	822 (129)	27	69.53

*Distinct letters indicate significant statistical differences (p< 0.05) depicted by Kaplan-Meier and Mantel-Cox (Log-rank) for fatigue tests.

**Table 4:** Survival rates, probability of specimens exceeding specified fatigue failure load and load (FFL) or cycles for failure (CFF) with their corresponding standard error measurements.

Groups	Fatigue Failure Load (N) / Cycles for Failure												
	200/ 10,000	300/ 20,000	400/ 30,000	500/ 40,000	600/ 50,000	700/ 60,000	800/ 80,000	900/ 90,000	1000/ 100,000	1100/ 110,000	1200/ 120,000	1300/ 130,000	1400/ 140,000
Picn	1	…	…	…	…	…	…	0.93 (0.06)	0.47 (0.13)	0.07 (0.06)	0	-	-
Ld	1	…	…	…	…	…	…	0.80 (0.10)	0.40 (0.13)	0.20 (0.10)	0.07 (0.06)	0	-
Yz	1	…	…	…	…	…	…	0.93 (0.06)	0.87 (0.09)	0.53 (0.13)	0.27 (0.11)	0.27 (0.11)	0
Peek	1	…	…	0.67 (0.12)	0.27 (0.11)	0.07 (0.06)	0	-	-	-	-	-	-

*The symbol ‘-’ denotes that no specimens were tested at the respective step.

** The symbol ‘…’ denotes absence of specimen failure on such step, maintaining the survival rate of the prior step.

**Figure 3:**
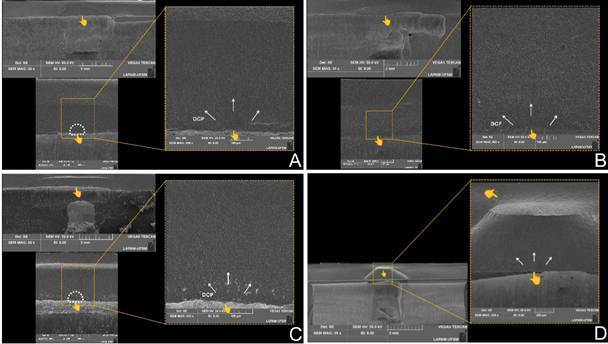
Representative SEM images of each group according to filled abutment material (A- Ld; B- Picn; C- Peek; D- Yz). Failure analysis showed that for Ld, Peek, and Picn groups, radial cracks were noted at the tensile side. However, both hertzian cone cracks and radial cracks were present for the Yz group, corroborating the occurrence of piston contact damage after higher load application.

The SEM images representing the failure pattern for each group are present in Figure 3. The failure analysis showed that for the Ld, Peek, and Picn groups, radial cracks were noted at the bonding surface of lithium disilicate restorative material (tensile side). However, for the Yz group, both Hertzian cone cracks and radial cracks were present, corroborating the occurrence of piston contact damage after higher load application.

## Discussion

Through the results, it is evident that the abutment material influenced the monotonic and fatigue failure load of the restoration in the context of implant-supported restorations. Thus, the null hypothesis was rejected.

Due to the friability of ceramic materials, the success of the restorative set depends on the interaction between the abutment material and the lithium disilicate restoration, particularly regarding the stress distribution and behavior when a load is applied ^21^. Although lithium disilicate restorations exhibit satisfactory mechanical strength, they are susceptible to failure under tensile stress ^14^. In this context, the choice of abutment material is crucial as it provides support and modulates the bending effect on the restorative material ^19^. In the present study, even with the screw access hole being filled with resin composite in all groups, it was evident that the abutment material continued to significantly influence the mechanical behavior of the restorative set due to its mechanical properties and support. Previous studies reported that such influence can be explained by the variation in the elastic modulus and microstructure among the materials ^14^ ^,^ ^19^ ^,^ ^21^, since materials that present a higher modulus are more rigid and mechanically resistant when under stress. Thus, the stiffer the substrate, the lower the bending effect expected for the lithium disilicate restoration ^32^.

Among the evaluated abutment materials, zirconia (Yz) enhanced the mechanical performance of lithium disilicate restorations compared to other materials, due to its high elastic modulus (GPa = 210). This property can be attributed to its polycrystalline microstructure, as the crystals provide greater mechanical strength and, consequently, a high stiffness ^33^. Hence, it is feasible to assume that zirconia abutments provide a supporting and reinforcing effect for lithium disilicate restoration, which is subjected to tensile stress at the bonding surface ^27^ ^,^ ^34^. This finding is corroborated by finite element analysis, which showed that zirconia mitigates the bending effect on lithium disilicate, resulting in higher stress concentration at the bottom surface of the restorative material (Figure 2). Besides, the SEM analysis showed that damages at the top surface of the specimens were found for the Yz group, as a response to the increased support for the bonding surface and higher stress concentration (Figure 3). Thus, for the Yz group, both radial and cone cracks were noted, harming the detection of the failure origin.

When lithium disilicate (Ld) and polymer-infiltrated ceramic network (PICN) abutments were used, intermediate fatigue failure load values were observed (Table 3), which were lower compared to Yz in this context. Despite the elastic modulus of Ld and Picn, which are around 95 GPa and 28 GPa, respectively ^14^ ^,^ ^18^ ^,^ ^31^, it is clear that the evaluated outcome was also influenced by the microstructural characteristics of these materials. Ld exhibits satisfactory mechanical strength because it is a glass-ceramic composed of a glass matrix reinforced with lithium disilicate crystals ^6^, making this material versatile in terms of aesthetics and resistance. Even so, this material presents less polycrystalline content when compared to zirconia, which may be attributed to the lower elastic modulus of the glass-ceramic. On the other hand, Picn is a hybrid material composed of a ceramic matrix infiltrated with a polymeric content, enhancing its fracture toughness ^17^ ^,^ ^18^. Thus, even presenting distinct compositions, depicted by the presence of crystalline and polymeric content, both materials promoted similar reinforcement effects and mechanical performance to the restorative lithium disilicate. The finite element analysis also corroborates such assumptions, since it showed similar levels of stress and bending effect of the restoration at the bonding interface, which was more pronounced when compared with Yz ^30^, as illustrated by the radial cracks detected in the failure analysis (Figure 3).

When the Peek abutment was used, the lowest mechanical performance was found for the restorative material. As mentioned, the bending effect in the restorative material varies according to the microstructure and elastic modulus of the abutment, so the less crystalline and more vitreous/polymeric the abutment material, the greater the bending effect and, consequently, the lower the mechanical performance of the restorative assembly. In this sense, Peek presents the lowest elastic modulus among the evaluated abutment materials (4.1 GPa) ^29^, while also presenting almost only a polymeric matrix in its composition, thus leading to the lowest load for the failure of the restorative lithium disilicate when bonded to this material. As a consequence, the stress concentration led to an increased bending effect in the bonding surface of this restorative set, as shown by the FEA analysis (Figure 2) and SEM images (Figure 3), thus abutments presenting more rigid microstructure and higher elastic modulus may be preferred for implant-supported restorations.

Despite the findings of the present study, some limitations should be mentioned. Since the specimens were fabricated in a simplified disc shape, their geometry does not produce exactly the same results as specimens with the shape of a dental crown, due to the anatomy of the cusps and their interaction with masticatory loads, which differ from the loads applied on flat surfaces. Another limitation is the absence of the screw itself, with only the access hole present, which can alter the stress distribution. Furthermore, the loads applied in this study were axial, and the screw-access hole was always at the center of the load application, which is different from the oral environment, where different angulations and oblique loads are also present, which may alter the influence of the screw-access hole presence in the abutment or the restorative set ^35^. However, the present in vitro study succeeded in showing that the microstructure of the evaluated abutment materials, despite the presence of a screw-access hole, plays an important role in the mechanical performance of the restorative set, which is absolutely important for the clinical scenario. Thus, when considering implant-supported restorations over screwed abutments, a critical evaluation of the mechanical properties of the available materials for aesthetic abutments must be taken by the clinicians, in order to promote longevity for the crown. Even so, it is essential that future studies on this topic include the mentioned limitation factors to provide a more clinically accurate response.

## Conclusion

The abutment material is crucial in determining the mechanical fatigue behavior of restorations in implant-supported prostheses with screw-retained abutments. This study found that zirconia is the most suitable material for optimizing the longevity of lithium disilicate restorations. In contrast, Peek presented the lower performance for screw-retained abutments due to increased stress concentration and reduced lifetime.

## Data Availability

The research data are available upon request.
